# MERS-CoV

**DOI:** 10.1186/1471-2334-14-S2-S22

**Published:** 2014-05-23

**Authors:** Marcel A Müller

**Affiliations:** 1 Institute of Virology, University of Bonn Medical Center, Bonn, 53113, Germany

## 

In 2002/03, a novel coronavirus (CoV) termed severe acute respiratory syndrome (SARS)-CoV emerged in China and caused an epidemic in which approximately 800 of 8000 cases died. Ten years later, a newly emerged Middle East respiratory syndrome (MERS)-CoV has been confirmed in 189 patients of which 82 had a fatal outcome. All MERS cases were linked to the Arabian Peninsula and to date thirteen cases were imported into the European Union and Africa. The clinical presentation resembles that of SARS-CoV as both viruses affect mainly the lower respiratory tract. As for other mammalian CoV, bats harbor a high diversity of related viruses and are suspected to be the animal reservoir. Recent studies have revealed that dromedary camels are the most likely intermediate host. MERS-CoV has been circulating in dromedary camels for decades raising the question whether earlier human cases may have remained undetected. Preliminary serological surveys in Saudi Arabia could not determine increased seroprevalence for MERS-CoV antibodies in the population. The possibility of human to human transmission has been demonstrated but is clearly limited. The dipeptidyl peptidase-4 (DPP4) was identified as a cellular receptor for MERS-CoV entry. DPP4 was found predominantly on non-ciliated bronchial epithelial and alveolar cells in the lower parts of the lungs. This might explain the lack of secondary infections because exposure to high doses of viruses may be necessary. *In-vitro* studies showed that MERS-CoV inhibited important cellular pathways in particular the interferon (IFN) response. Confirmatory *in-vivo* experiments are still limited due to the lack of a suitable animal model. Non-human primates were shown to be susceptible but did not develop a disease to an extent as humans. A combination of pegylated IFN-alpha and ribavirin improved the clinical symptoms confirming previous *in-vitro* experiments. The development of vaccines is ongoing and vaccination programs in dromedary camels might be the optimal choice to control the current outbreak and prevent future re-introductions into the human population.

